# Features of Tat Protein in HIV-1 Sub-Subtype A6 Variants Circulating in the Moscow Region, Russia

**DOI:** 10.3390/v15112212

**Published:** 2023-11-04

**Authors:** Anna Kuznetsova, Kristina Kim, Alexander Tumanov, Iana Munchak, Anastasiia Antonova, Aleksey Lebedev, Ekaterina Ozhmegova, Elena Orlova-Morozova, Elena Drobyshevskaya, Alexander Pronin, Aleksey Prilipov, Elena Kazennova

**Affiliations:** 1 Gamaleya National Research Center for Epidemiology and Microbiology, 123098 Moscow, Russia; kimsya99@gmail.com (K.K.); desep@mail.ru (A.T.); yana_munchak@mail.ru (I.M.); anastaseika95@mail.ru (A.A.); lebedevalesha236@gmail.com (A.L.); belokopytova.01@mail.ru (E.O.); a_prilipov@mail.ru (A.P.); kazennova@rambler.ru (E.K.); 2 Mechnikov Scientific Research Institute of Vaccines and Serums, 105064 Moscow, Russia; 3 Moscow Regional Center for the Prevention and Control of AIDS and Infectious Diseases, 129110 Moscow, Russia; orlovamorozova@gmail.com (E.O.-M.); elena_dr@bk.ru (E.D.); alexanderp909@gmail.com (A.P.)

**Keywords:** HIV-1, sub-subtype A6, Tat, Moscow Region, Russia

## Abstract

Tat, the trans-activator of transcription, is a multifunctional HIV-1 protein that can induce chronic inflammation and the development of somatic diseases in HIV-infected patients. Natural polymorphisms in Tat can impact the propagation of the inflammatory signal. Currently, Tat is considered an object for creating new therapeutic agents. Therefore, the identification of Tat protein features in various HIV-1 variants is a relevant task. The purpose of the study was to characterize the genetic variations of Tat-A6 in virus variants circulating in the Moscow Region. The authors analyzed 252 clinical samples from people living with HIV (PLWH) with different stages of HIV infection. Nested PCR for two fragments (*tat1*, *tat2*) with subsequent sequencing, subtyping, and statistical analysis was conducted. The authors received 252 sequences for *tat1* and 189 for *tat2.* HIV-1 sub-subtype A6 was identified in 250 samples. The received results indicated the features of Tat1-A6 in variants of viruses circulating in the Moscow Region. In PLWH with different stages of HIV infection, C31S in Tat1-A6 was detected with different occurrence rates. It was demonstrated that Tat2-A6, instead of a functional significant ^78^RGD^80^ motif, had a ^78^QRD^80^ motif. Herewith, G79R in Tat2-A6 was defined as characteristic amino acid substitution for sub-subtype A6. Tat2-A6 in variants of viruses circulating in the Moscow Region demonstrated high conservatism.

## 1. Introduction

The human immunodeficiency virus (HIV) Tat (the trans-activator of transcription) protein is a non-structural regulatory HIV protein which modulates the activity of the viral promoter [1]. Moreover, Tat is released by HIV-infected cells in the surrounding microenvironment and then can be taken up by other HIV-infected cells, as well as uninfected cells. Herewith, latently HIV-infected cells can be reactivated by Tat. In uninfected cells, Tat can induce apoptosis, the release of neurotransmitters, oxidative stress, and inflammation [2,3]. Chronic inflammation modulated by Tat in people with HIV leads to several comorbidities such as HIV-associated neurocognitive diseases and cardiovascular impairment [2].

Tat is coded by two exons and contains six different functional domains. The first five domains, Tat1 (1 to 72 amino acids, a. a.), are encoded by the first exon (Figure S1). The N-terminal domain, or the acidic domain (1–21 a. a.), is a proline-rich region with conserved tryptophan residue in the 11 position. The second domain (22–37 a. a.), or cysteine-rich region, contains a highly conserved cysteine-rich tract. The third domain, or a hydrophobic core region, includes 38–48 a. a. The fourth, or the basic domain (49–57 a. a.), is composed of a well-conserved arginine-rich motif. The fifth domain (58–72 a. a.), or a glutamine-rich region, has the greatest genetic variability. The first five domains are involved in Tat transactivation activity, in Tat nuclear localization, and in Tat uptake by other cells. The sixth domain, Tat2 (73 to 101 a. a.), is encoded by the second exon and, as it was supposed, may contribute to viral infectivity and binding to cell-surface integrins [1,4].

The effect of natural amino acids substitutions in Tat on its functions and pathogenesis is a subject of much debate. Thus, C31S is a well-known natural polymorphism in Tat-C [5,6]. The experiments in cell cultures demonstrated that Tat-B and Tat-C induce different neuronal damage which could be associated with C31S [7]. The analysis of clinical HIV isolates clade C showed different neurovirulence for C30- and C30S-containing variants, and the authors supposed that it may be possible to predict neurocognitive outcomes for PLWH based on Tat genotyping [8]. Recently conducted in vitro experiments indicated that naturally occurring polymorphism in Tat basic domain R57S inhibits Tat uptake by bystander cells and reduces neuroinflammation. Also in this work, it was reported that R57G significantly decreased Tat basic domain uptake. Significant differences in occurrence of these polymorphisms in various subtypes were detected. Thus, in clade B in the 57 Tat position R was detected in 93.3% cases, whereas, in subtype C R57S was presented in 81.6% cases, and, in clade A and G R57G was identified in 71.2% and 75% cases, respectively [9]. Moreover, the comparison of HIV-1 Tat1-C during primary and chronic infection made it possible to identify seven sites with positive selection pressure and showed that the amino acid substitutions in these sites had significant functional consequences for Tat transactivation [10]. Thus, the comparison of clinical information from HIV-infected patients with Tat sequences could help to determine the correlation between the genetic diversity of Tat protein and HIV pathogenesis.

Currently, Tat is also a target for the development of new antiretroviral drugs and therapeutic vaccines [11]. The genetic variation of Tat protein in circulating HIV-1 variants has been the subject of many studies [6,12,13]. Herewith, it was shown that the virus of the HIV-1 C variant in a specific region could have its own unique signatures [14]. Therefore, the studying of Tat genetic diversity in virus variants circulating in separate regions is an interesting task.

The predominant HIV-1 variant in Russia now remains sub-subtype A6 [15,16], with the exception of in Siberia, Kaliningrad, and Vologda Regions, where CRF063_02A1 [17], CRF 03AB, and various unique recombinant forms (URFs) between subtypes A and B [18,19] are highly prevalent. Previously, we have identified characteristic features for Tat1-A6 by analyzing the sequences downloaded from the Los Alamos international database [20].

The aim of this study was to characterize the genetic variations of TatA6 in variants circulating in the Moscow Region, to evaluate sequence conservation of Tat1-A6 in the Moscow Region, to compare the genetic diversity of Tat1-A6 in HIV-infected patients in different stages of HIV-1 infection, and to identify characteristic features for Tat2-A6.

## 2. Materials and Methods

### 2.1. Study Population

Single peripheral blood samples were collected from 252 HIV-1-infected patients attending the Moscow Regional Center for the Prevention and Control of AIDS and Infectious Diseases between August 2019 and July 2020, during the implementation of the CARE project (https://www.careresearch.eu/ accessed on 24 September 2023). Three criteria for the formation of the study population were applied: (1) a well-documented history of HIV infection in patient, (2) permanent residency in the Moscow Region, and (3) informed consent for the research. Patients were not receiving antiretroviral therapy at the time of blood sampling. The protocol of the study was approved by the Committee on Biomedical Ethics of the D.I. Ivanovsky Institute of Virology of FSBI “National Research Center for Epidemiology and Microbiology named after Honorary Academician N.F. Gamaleya”, 123098, Moscow, Russia (protocol no. 16 of 8 February 2019).

Patients enrolled in the study were in stages II, III, and IV of HIV infection according to the Clinical Guidelines of the Ministry of Health of the Russian Federation [21] (Table 1). According to the current classification of HIV infection in Russia, the stage of the disease is established only on the basis of clinical manifestations wherein the level of viral load and CD4 is not a criterion for determining the clinical stage. Thus, the second stage of HIV infection, or stage of initial manifestations, usually is observed during the 12 months after seroconversion. The third stage of HIV infection, or subclinical stage, is characterized by slow progression of HIV infection, compensated by immune response modification. The only clinical manifestation of the disease at this stage is the increase in lymph nodes. On average, the third stage of HIV infection without antiretroviral therapy processes during 6–7 years. The fourth stage of HIV infection, or the stage of secondary manifestations, is characterized by the development of opportunistic infectious and/or oncological diseases [21].

### 2.2. DNA Extraction, Nested PCR, and Sequencing

Genomic DNA from whole blood was extracted using the simple salting-out procedure [22]. HIV-1 *tat exon 1* and *exon 2* were amplified using nested polymerase chain reaction (PCR) followed by double pass population sequencing. For *tat exon 1*, the first round of PCR was carried out using the following external primers: TatRevF1 (5′–CAGAATTGGGTGCCAACATAGCAG-3′) and Vpu1o (5′–GCCCAGACATTGTGTACTTCTTTATC-3′). Thereafter, an approximately 658 bp fragment containing *tat exon 1* was amplified with internal primers TatRevf2_v1 (5′–GTGCCAACATAGCAGAATAGGC-3′) and Vpu2o (5′GCATCTCTCCACACAGGTAC-3′). For tat *exon 2*, the first round of PCR was carried out using the following external primers: T2R1p (5′-CCAGCAGGAAAAGAATGAACAAG-3′) and T2R1o (5′-TGTCTGATTCTCCTAGGTAT-3′). Then, the fragment containing *tat* exon 2 (~593bp) was amplified with internal primers T2R2p (5′-AGGCAGGGATACTCACCC-3′) and T2R2o (5′ATAACCCTATCTGTCCCTTCAGCTAC-3′). The cycling conditions were the same for *tat exon 1* and *exon 2*. For the first round, there was 1 cycle at 95 °C for 5 min, 35 cycles at 95 °C for 30 s, 57 °C for 1 min, and 72 °C for 1 min 20 s, with a final extension at 72 °C for 10 min. For the second round, there was 1 cycle at 95 °C for 5 min, 35 cycles at 95 °C for 30 s, 53 °C for 1 min, and 72 °C for 1 min 20 s, with a final extension at 72 °C for 10 min. Purified PCR products were sequenced using the primers TatRevf2_v1 and Vpu2o for *tat exon 1* and T2R2p and T2R2o for *tat exon 2*. The sequences thus generated were manually edited in SeqMan II 6.1.

### 2.3. HIV-1 Subtyping

Originally, subtyping of HIV-1 was performed on *tat exon 1* by two methods: (1) COMET HIV-1 (http://comet.retrovirology.lu/, accessed on 15 September 2023), which uses context-based modeling for rapid typing of HIV-1 viruses; (2) Recombinant Identification Program version 3 (RIP 3.0) with a window size of 50 nucleotides, which detects recombination in shorter sequences (<700 nt) (https://www.hiv.lanl.gov/content/sequence/RIP/RIPexplain.html, accessed on 15 September 2023). Phylogenetic analysis was performed to clarify the results of primary subtyping.

Pairwise and multiple alignments of nucleotide sequences were performed using the AliView program algorithm [23]. The total length of the alignment was 215 bp (positions 5831–6045 based on the numbering of the reference strain HXB2, GenBank no. K03455). Phylogenetic analysis was performed by the maximum likelihood method using the IQ-TREE program [24]. The source of the reference sequences was the database of the Los Alamos Institute, USA (https://www.hiv.lanl.gov/components/sequence/HIV/search/search.html, accessed on 15 September 2023). As a nucleotide substitution model, the GTR + I + G4 model was used, the choice in favor of which was made on the basis of the Akaike information criterion in jModelTest v. 2.1.7 [25]. The reliability of the resulting phylogenies was assessed using the bootstrap test and the Shimodaira–Hasegawa approximate likelihood ratio test (SH-aLRT) of 1000 post-start iterations. Clusters with SH-aLRT support greater than 0.9 were considered to be reliably established. Visualization and graphic processing of trees were carried out in the FigTree v.1.4 and iTOL programs [26]. In further analysis, HIV-1 sub-subtype A6 sequences were included only.

### 2.4. Tat1-A6 Sequences Conservation in the Moscow Region

For evaluating the sequence conservation of Tat1-A6 in virus variants circulating in the Moscow Region, the conservation of amino acid residues in Tat1-A6 was compared to the reference group. To form a reference group from the HIV-1 Los Alamos database (www.hiv.lanl.gov, accessed on 15 September 2023), 141 sequences of Tat1-A6 (accessed on 22 July 2022) were downloaded. Herewith, the duplicate sequences from the same subject were excluded from the analysis. The consensus sequence in the reference group was used as a reference sequence. Sequence conservation was understood as the frequency of occurrence of an amino acid, which was characteristic for the reference sequence, in the analyzed group. With the use of the Nonparametric Statistics software module from the STATISTICA 8.0 package (StatSoft Inc., Tulsa, OK, USA), the sites with statistically significant differences were identified (*p* < 0.05 when using the χ^2^ criterion). 

### 2.5. Genetic Diversity of Tat1-A6 in PLWH with Different Stages of Infection

To compare the genetic diversity of Tat1-A6 in PLWH with different stages of HIV-1 infection, the received Tat1-A6 sequences were grouped according to the stage of the patient’s disease. First, in MEGA v.10.2.2, in each group of sequences, natural polymorphisms were detected with respect to the reference strain HXB2 (K03455). Herewith, the polymorphisms were understood as mutations–single substitutions occurring in ≥1% of cases [27]. With the use of the Nonparametric Statistics software module from the STATISTICA 8.0 package (StatSoft Inc., Tulsa, OK, USA), the sites with statistically significant differences were identified (*p* < 0.05 when using the χ^2^ criterion) between the groups.

### 2.6. Characteristic Features for Tat2-A6 in the Moscow Region

The analysis of Tat2-A6 characteristic features in the Moscow Region was carried out in two stages. Since, previously, the analysis of Tat2-A6 characteristics was not carried out, first, a comparative analysis of Tat2-A6 with the most genetically close Tat2-A1, the most studied Tat2-B, and the most widespread Tat2-C was conducted. For this purpose, from the international Los Alamos database (Los Alamos National Laboratory; www.lanl.gov, accessed on 15 September 2023), 205 Tat2-A6 sequences, 236 Tat2-A1 sequences, 250 Tat2-B sequences, and 250 Tat2-C sequences were selected (accessed on 1 July 2023). Herewith, the duplicate sequences from the same subject and the sequences of Tat2-A6 from the Moscow Region were excluded from the analysis. Initially, a visual comparison of amino acid sequences between different Tat2 groups was made based on analyzing graphical representations in the Weblogo program (https://weblogo.berkeley.edu/logo.cgi, accessed on 15 September 2023). Then, the natural polymorphisms into Tat2 functionally significant motifs, RGD and ESKKKVE (Figure S1), were compared between A6 and other clades [4]. For each clade, polymorphisms regarding the consensus sequence of clade B were detected. The sites with statistically significant differences (*p* < 0.05 when using the χ^2^ criterion) in occurrence between A6 and A1, A6 and B, A6 and C were identified using the Nonparametric Statistics software module from the STATISTICA 8.0 package (StatSoft Inc., Tulsa, OK, USA).

At the second stage, the conservation of Tat2-A6 sequences in virus variants circulating in the Moscow Region was compared with the reference group which was formed by Tat2-A6 sequences loaded from the Los Alamos database. The consensus sequence in the reference group was used as a reference sequence. Sequence conservation was understood as the frequency of occurrence of an amino acid, which was characteristic for the reference sequence, in the analyzed group. With the use of the Nonparametric Statistics software module from the STATISTICA 8.0 package (StatSoft Inc., Tulsa, OK, USA), the sites with statistically significant differences were identified (*p* < 0.05 when using the χ^2^ criterion).

## 3. Results

### 3.1. Sequencing Results

For all patients enrolled in the study (252), *tat 1* sequences were received. Additionally, for 35 patients in the second stage of HIV infection, for 71 patients in the third stage, and for 83 patients in the fourth stage, *tat 2* sequences were received (189 in total). All received HIV-1 sequences were submitted to GenBank with the accession numbers for *tat 1:* OR460390–460641, for *tat 2*: OR460201–OR460389.

### 3.2. HIV-1 Subtyping

**Figure 1:**
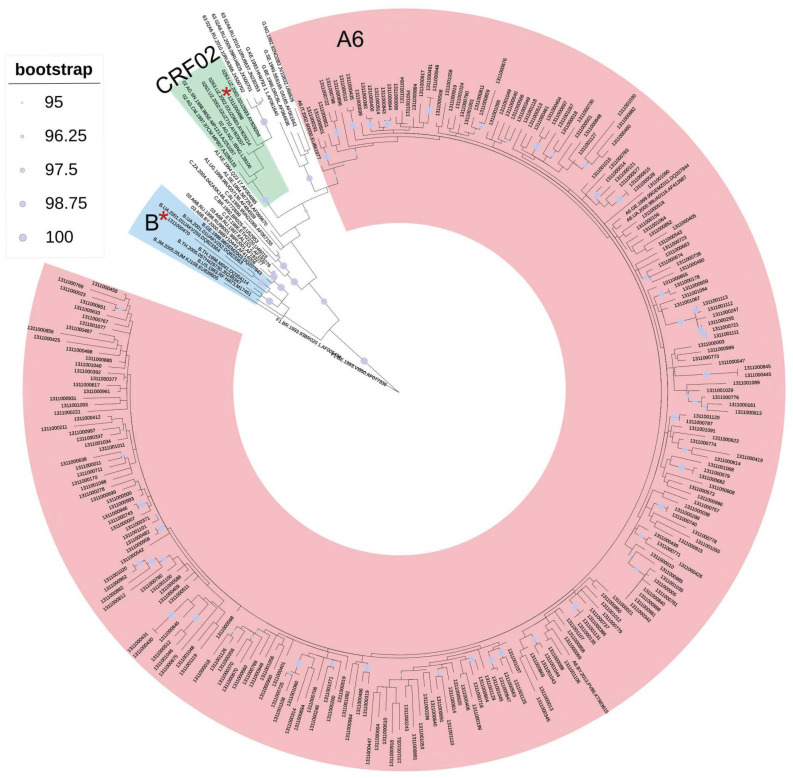
Phylogenetic tree of 252 HIV-1 sequences of the tat gene obtained from infected individuals from the Moscow Region in 2019–2020. Asterisks mark the positions of the studied sequences belonging to the B and CRF02_AG genetic variants. *Note*: Numbers of root reference sequences (F1): F1.BR.1993.93BR020 1.AF005494 and F1.BE.1993.VI850.AF077336.

The results of primary subtyping and phylogenetic analysis completely coincided and showed the following correlation of genetic variants about the *tat* gene: sub-subtype A6—99.2% (250/252), subtype B—0.4% (1/252), and CRF02_AG—0.4% (1/252) (Figure 1). Further, only the sequences of the sub-subtype A6 were analyzed.

### 3.3. Tat1-A6 Sequences Conservation in the Moscow Region

During the comparison of Tat1-A6 sequences conservation in the group of virus variants circulating in the Moscow Region to the reference group, in 11 sites, the sequence conservation of Tat1-A6 in the Moscow Region was less than in the reference group with statistically significant differences (*p* < 0.05): 2D, 7N, 21A, 23S, 31C, 47Y, 54H, 57G, 61S, 62S, 68P (Table S1). After Bonferroni correction (*p* < 0.0045), seven sites were significant: 2D, 23S, 31C, 54H, 57G, 61S, 62S (Table S1).

Two sequences in the group of sequences from the Moscow Region contained insertions. The 1311001071 sequence contained proline insertion between 53 and 54 amino acids (53–54insP). The 1311000200 sequence contained threonine insertion between 57 and 58 amino acids (57–58insT).

### 3.4. Genetic Diversity of Tat1-A6 in HIV-Infected Patients at Different Stages of Infection

During the comparison of the genetic diversity of Tat1-A6 in PLWH with different stages of infection, 13 sites with statistically significant differences were detected (Table 2).

### 3.5. Characteristic Features for Tat2-A6 in the Moscow Region

**Figure 2:**
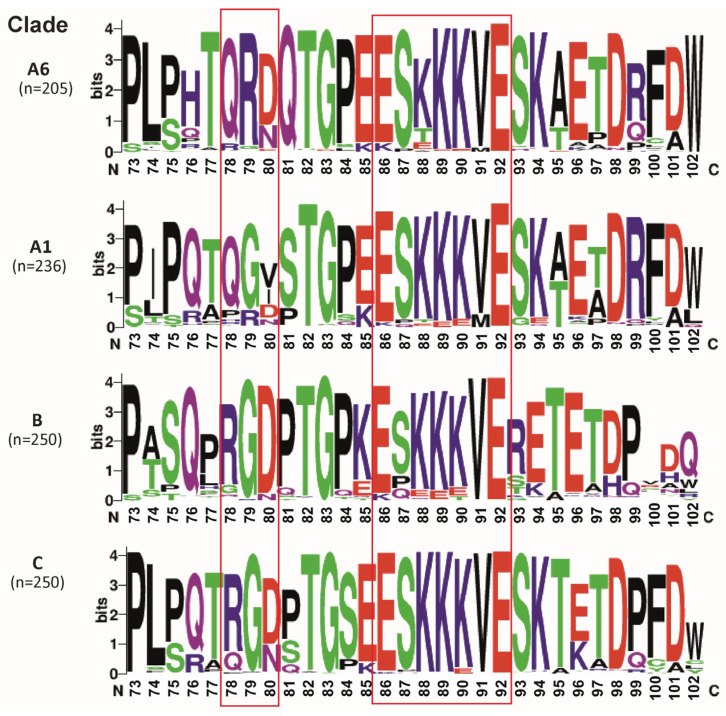
Different HIV-1 clades display variation in Tat2. RGD and ESKKKVE functionally significant domains are shown in bold. *Note*: Visualization was made in weblogo.berkeley.edu.

The visual comparison of amino acid sequences in Tat2-A6, Tat2-A1, Tat2-B, and Tat2-C groups demonstrated variability between groups (Figure 2). 

The subsequent statistically analysis focused only on the two functionally significant domains (RGB and ESKKKVE), which are expected to have a higher conservation.

Twelve sites with significant differences (*p* < 0.05) in occurrence between Tat2-A6 and Tat2-A1 were detected: R78Q, R78P, R78H, G79R, D80N, D80V, D80I, S87Q, K88T, K88E, K90E, V91M (Table S2). After Bonferroni correction (*p* < 0.0042), eight sites were significant: R78Q, R78P, G79R, D80N, D80V, D80I, K88T, V91M (Table S2).

Fourteen sites with significant differences (*p* < 0.05) in occurrence between Tat2-A6 and Tat2-B were detected: R78Q, R78G, G79R, D80N, D80E, S87P, S87Q, K88Q, K88T, K88A, K89E, K90E, K90T, V91M (Table S2). After Bonferroni correction (*p* < 0.0036) eight sites were significant: R78Q, R78G, G79R, D80N, S87P, S87Q, K88T, K89E (Table S2).

Eight sites with significant differences (*p* < 0.05) in occurrence between Tat2-A6 and Tat2-C were detected: R78Q, G79R, D80E, E86K, K88T, K88A, K88E, V91M (Table S2). After Bonferroni correction (*p* < 0.0062), five sites were significant: R78Q, G79R, E86K, K88T, K88E (Table S2).

During the comparison of Tat2-A6 sequences conservation in the group of virus variants circulating in the Moscow Region to the reference group, four sites with statistically significant differences (*p* < 0.05) were detected: 73P, 86E, 97T, 101D (Table S3). After Bonferroni correction (*p* < 0.0125), only one site was significant—73P, in which conservation of the sequences in the group of virus variants circulating in the Moscow Region was higher than in the reference group (Table S3).

## 4. Discussion

Today, HIV infection is treated with combined antiretroviral therapy (cART), which involves lifelong use of antiretroviral drugs, whereas an HIV functional cure characterized by sustained viral suppression without the need for lifelong medications has not been developed. The problems of HIV drug resistance, drug toxicity, and drug–drug interactions form the need for regular changes in cART regimens in patients’ treatment and, as a result, create a background for creating new treatment approaches. Currently, based on Tat protein, different medications are being developed. Thus, two molecules, didehydro-cortistatin A (dCA) and triptolide, inhibit the activity of Tat protein by directly affecting it and can be a basis for the development of a new class of antiretroviral drugs, Tat inhibitors [28,29]. Long-term research has shown that Tat immunization induces progressive immune restoration and reduction of virus reservoirs and can represent an optimal vaccine candidate for functional cure and eradication strategies [30]. The investigation of Tat genetic diversity can have an influence on vaccine formulations and can have implications for the development of antiretroviral drugs.

Natural polymorphisms in Tat can have a potential effect on neuropathophysiology and clinical outcomes [4,9]. Thus, the study of natural polymorphism in HIV-1 Tat protein in different subtypes, in virus variants circulating in different territories, and in patients at different stages of HIV infection may allow identification of the groups of PLWH with increased risk of comorbid diseases, correction of ART in risk groups, and delay of the occurrence of comorbid diseases. This is the first study of genetic variation of Tat protein in HIV-1 sub-subtype A6 variants circulating in Russia in the Moscow Region.

The genotyping of the virus in clinical samples was carried out based on the analysis of *tat 1* nucleotide sequences. In 250/252 clinical samples, the virus sub-subtype A6 was identified, in 1/252—subtype B, in 1/252—CRF02_AG. These results are consistent with the data that show subtype A6 is still the major HIV-1 subtype in Central Russia and the Moscow Region in particular [16,31].

Seven sites were detected in which the sequence conservation of Tat1-A6 in the Moscow Region was less than in the reference group, and some of these sites are located in function cure regions. Thus, cysteine in 31 position is needed for Tat to inhibit membrane traffic processes, neurosecretion, and phagocytosis in uninfected cells [32]. It was shown that substitution of cysteine for serine in the 31 position attenuates monocyte chemotactic function without modulating transactivation property [5,33]. 54H and 57G are placed in a well-conserved 49RKKRRQRRR57 motif in the fourth domain, which is a positively charged region and is the transactivation response element (TAR) binding region [34]. 62S is in the fifth, glutamine-rich domain, in the positions involved in phosphorylation, which helps to increase the interaction with the TAR element and enhances viral transcription [12,35]. Separately, it should be noted that, in two Tat1-A6 sequences in the Moscow Region, amino acid inserts were identified: in 1311001071—53–54insP, in 1311000200—57–58insT. The functional meaning of these inserts is unknown, but they are located in the conserved 49RKKRRQRRR57 motif and can possibly influence Tat interaction with the TAR element on newly synthesized HIV RNA and, as a result, on the transactivation of transcription. Generally, the sequence conservation of Tat1-A6 in the Moscow Region in some sites, even in sites located in well-conserved regions, is less than in the reference group, which may potentially influence the efficiency of Tat-based therapeutic agents in the future. These findings also indicate the need to research Tat1-A6 genetic diversity in different regions.

When comparing Tat1-A6 genetic diversity in PLWH with different stages of HIV-1 infection, a C31S substitution was detected with a statistically significant difference in occurrence after Bonferroni correction (Table 2). C31S has functional significance; it significantly reduces the pro-inflammatory function and neurotoxicity of Tat [9]. In this study, the C31S substitution was statistically more common in patients at the second stage of HIV infection (29.2%) than in patients with the third (9.1%) and with the fourth stages (4.8%). The result obtained can be associated with various A6 variants circulating at different periods in the Moscow Region, as well as with the features of HIV-1 variants circulating in different stages of the disease. To clarify this question, it is necessary to monitor the modification of TatA6 in the same patients in different stages of HIV infection.

Currently, Tat2 is much less studied than Tat1. It has been shown that Tat2 reduces innate immune responses and has two functionally significant motifs: RGD and ESKKKVE [4,36]. Thus, the RGD motif is a ligand for several integrins, whereas the ESKKKVE motif can possibly be involved in optimal HIV-1 replication in vivo and in delaying apoptosis in CD4 + T lymphocytes [4,37]. Since, at this moment, the analysis of Tat2-A6 characteristics has not been carried out, initially the comparison of Tat2-A6 with the most genetically close Tat2-A1, the most studied Tat2-B, and the most widespread Tat2-C was conducted. The results obtained showed a significant variation between clades. The comparative analysis of natural polymorphisms in functionally significant motifs of Tat2 defined that Tat2-A6 and Tat2-A1 clades instead of an ^78^RGD^80^ motif had ^78^QRD^80^ and ^78^QGV^80^ motifs, respectively, and G79R can be marked as a characteristic substitution for Tat2-A6. Moreover, in the ^86^ESKKKVE^92^ motif, Tat2-A6 has the high variability in position 88, which was significantly different from Tat2-A1, Tat2-B, and Tat2-C.

Only in one site, P73, the sequence conservation of Tat2-A6 in the Moscow Region was different, higher, compared to the sequence conservation of Tat2-A6 in the reference group, which can possibly be explained by the founder effect. 

## 5. Conclusions

The results of the study indicated the features of Tat1-A6 in variants of viruses circulating in the Moscow Region; sites with reduced conservatism were detected in well-conserved functional significant regions. It was identified that in Tat1-A6, the functional significant substitution C31S had a statistically significant difference in occurrence in PLWH with different stages of HIV infection. A limitation of this study was the analysis of different patients at different stages of the disease. Therefore, these findings require confirmation in the evaluation of Tat1 polymorphisms in the same patients at different stages of HIV infection. It was shown that Tat2-A6, instead of a functional significant ^78^RGD^80^ motif, had a ^78^QRD^80^ motif. Thus, G79R was specified as a characteristic amino acid substitution for Tat2-A6. In general, Tat2-A6 in virus variants circulating in the Moscow Region demonstrated higher conservatism than Tat2-A6 in the reference group, which can possibly be explained by the founder effect.

## Figures and Tables

**Table 1:** General characteristics of HIV-1-infected patients from the Moscow Region classified based on the stages of HIV infection.

Characteristics	Stage of Initial Manifestations/Stage II	Subclinical Stage/Stage III	Stage of Secondary Manifestations/Stage IV
No. of patients	49	100	103
Age (years)	38.7 (18–62)	40.5 (20–70)	41.4 (24–64)
Sex: Male Female	29 20	53 47	75 28
Viral load (log_10_ no. of RNA copies/mL)	5.1 (3.1–7)	4.7 (3.3–6.2)	5.1 (3.6–6.4)
CD4 count (no. of cells/µL)	592 (108–1062)	427.7 (65–1658)	236.1 (8–823)

**Table 2:** The mutations in Tat1-A6 with statistically significant differences in occurrence in the groups of HIV-1- infected patients at different stages of HIV infection *.

Mutation	II N = 48	III N = 99	IV N = 103	*p* II–III	*p* III–IV	*p* II–IV
T23S	29	76	76	0.0396	-	-
K29I	6	9	2	-	0.0252	0.0070
C31S	14	9	5	**0.0017**	-	**<0.0001**
V36M	8	7	6	-	-	0.0325
R53K	6	7	3	-	-	0.0205
R53S	2	0	2	0.0409	-	-
Q54S	4	1	5	0.0216	-	-
R57G	35	86	81	0.0376	-	-
R57E	2	0	0	0.0409	-	0.0370
Q60L	2	1	0	-	-	0.0370
Q60C	2	1	0	-	-	0.0370
N61G	3	4	0	-	0.0394	0.0104
S70P	9	7	15	0.0330	-	-

* Note. *p*-values are given for the positions with *p* < 0.05; the positions with *p* ≥ 0.05 are marked “-”. In bold: significant in the χ^2^ test with Bonferroni correction *p* < 0.0017.

## Data Availability

The data presented in this study are available on request from the corresponding author. The data are not publicly available due to the privacy policy of the Russian HIV database.

## Supplementary Materials

The following supporting information can be downloaded at: https://www.mdpi.com/article/10.3390/v15112212/s1, Figure S1: *tat* gene and functional domains of Tat protein; Table S1: The amino acid positions in Tat1-A6 with a statistically significant difference in the frequency of conservation between the sequences in the group of virus variants circulating in the Moscow Region and in the reference group (the sequences downloaded from the Los Alamos database); Table S2: Natural polymorphisms into Tat2 functionally significant motifs, ^78^RGD^80^ and ^86^ESKKKVE^92^, in different clades; Table S3: The amino acid positions in Tat2-A6 with statistically significant differences in the frequency of conservation between the sequences in the group of virus variants circulating in the Moscow Region and in the reference group (the sequences downloaded from the Los Alamos database).
