# Supplementary material for: Features of Tat Protein in HIV-1 Sub-Subtype A6 Variants Circulating in the Moscow Region, Russia
Source: Viruses. 2023 Nov 4;15(11):2212. doi: 10.3390/v15112212 (PMC10675479; doi:10.3390/v15112212)
Supplement: Supplementary file 1 [file viruses-15-02212-s001.zip › viruses-2661059-supplementary.pdf]

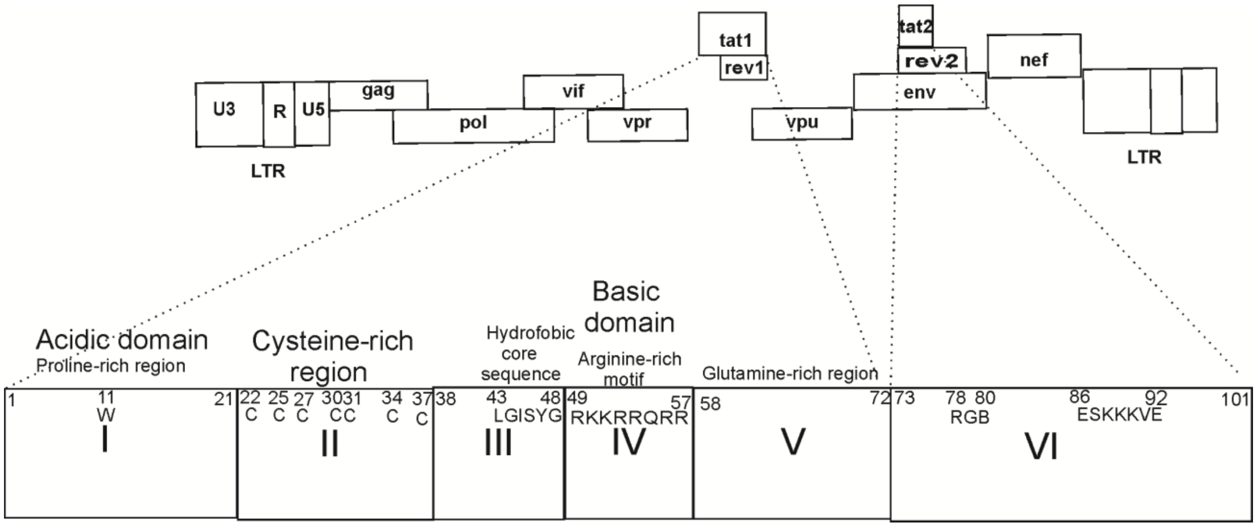

Figure S1. *tat* gene and functional domains of Tat protein. Tat is encoded by two exons. The first five domains are encoded by the first exon. I - first domain, proline-rich region: in 11 position - conserved tryptophan residue (W); II - second domain, cysteine-rich region: 7 highly conserved cysteine residues – in 22, 25, 27, 30, 31, 34 and 37 positions; III - third domain: contains hydrophobic core sequence ^{43}LGISYG^{48}; IV - fourth domain, arginine-rich region, binds to the TAR element of HIV RNA; V - fifth domain, glutamine-rich region. The sixth domain is encoded by the second exon: contains ^{78}RGB^{80} and ^{86}ESKKKVE^{92} functionally significant motifs.

Table S1. The amino acid positions in Tat1-A6 with a statistically significant difference in the frequency of conservation between the sequences in the group of virus variants circulating in the Moscow Region and in the reference group (the sequences downloaded from the Los Alamos database).

**Amino acid***	**Reference** group N=141 (100%)**	**Moscow Region N=250 (100%)**	***p***
2D	140 (99.3%)	225 (90%)	**0.0004**
7N	132 (93.6%)	216 (86.4%)	0.0285
21A	140 (99.3%)	238 (95.2%)	0.0303
23S	121 (85.8%)	181 (72.4%)	**0.0024**
31C	129 (91.5%)	197 (78.8%)	**0.0012**
47Y	141 (100%)	239 (95.6%)	0.0115
54H	89 (63.1%)	108 (43.2%)	**0.0002**
57G	130 (92.2%)	202 (80.8%)	**0.0025**
61S	119 (84.4%)	172 (68.8%)	**0.0007**
62S	121 (85.8%)	167 (66.8%)	**<0.0001**
68P	84 (59.6%)	118 (47.2%)	0.0187

*- the amino acid in the reference sequence. Consensus sequence in reference group was used as a reference sequence; ** – the group of Tat1-A6 sequences downloaded from the HIV-1 Los Alamos Database (www.hiv.lanl.gov). In bold: significant in the χ^2 test after Bonferroni correction $p < 0.0045$

Table S2. Natural polymorphisms into Tat2 functionally significant motifs, ^{78}RGD^{80} and ^{86}ESKKKVE^{92}, in different clades*.

**Domain**	**Substitution**	**A6**<br>N=205	**A1**<br>N=236	**B**<br>N=25<br>0	**C**<br>N=250	***p***<br>A6-A1	***p***<br>A6-B	***p***<br>A6-C
	R78Q	186	190	8	50	**0.0025**	**<0.0001**	**<0.0001**
	R78P	3	22	2	5	**0.0004**	-	-
	R78H	1	8	4	0	0.0316	-	-
	R78G	0	0	23	0	-	**<0.0001**	-
**RGD**	G79R	194	38	4	4	**<0.0001**	**<0.0001**	**<0.0001**
	D80N	45	25	16	74	**0.0011**	**<0.0001**	-
	D80E	6	3	0	0	-	0.0065	0.0065
	D80V	1	94	0	2	**<0.0001**	-	-
	D80I	0	57	0	2	**<0.0001**	-	-
	E86K	15	10	16	5	-	-	**0.0059**
	S87P	7	10	35	6	-	**0.0001**	-
	S87Q	0	8	31	3	0.0078	**<0.0001**	-
	K88Q	0	0	5	1	-	0.0418	-
	K88T	30	8	3	1	**<0.0001**	**<0.0001**	**<0.0001**
**ESKKKVE**	K88A	5	2	0	0	-	0.0130	0.0130
	K88E	16	7	17	1	0.0226	-	**<0.0001**
	K89E	4	13	21	1	-	**0.0027**	-
	K90E	5	17	18	13	0.0219	0.0211	-
	K90T	1	1	11	1	-	0.0096	-
	V91M	9	30	2	2	**0.0021**	0.0131	0.0131

***Note.** Amino acid substitutions were defined as the substitutions in the indicated positions in comparison with the consensus sequence of clade B. p values are given for the positions with $p < 0.05$, the positions with $p \geq 0.05$ are marked "-". In bold: significant in the χ^2 test with Bonferroni correction: A6-A1 $p < 0.0042$; A6-B $p < 0.0036$; A6-C $p < 0.0062$

Table S3. The amino acid positions in Tat2-A6 with statistically significant differences in the frequency of conservation between the sequences in the group of virus variants circulating in the Moscow Region and in the reference group (the sequences downloaded from the Los Alamos database).

**Amino acid***	**Reference group** N=205 (100%)**	**Moscow Region N=189 (100%)**	***p***
73P	187 (91.2%)	186 (98.4%)	**0.0015**
86E	184 (89.8%)	180 (95.2%)	0.0404
97T	157 (76.6%)	128 (67.7%)	0.0495
101D	157 (76.6%)	160 (84.6%)	0.0436

*- the amino acid in the reference sequence. Consensus sequence in reference group was used as a reference sequence; ** – the group of Tat2-A6 sequences downloaded from the HIV-1 Los Alamos Database (www.hiv.lanl.gov). In bold: significant in the χ^2 test with Bonferroni correction $p < 0.0125$
